# 

**Published:** 1843-01-21

**Authors:** 


### INDEX TO VOLUME VI.

A.

Abscess, Dr. Tucker's case of, 74; iliac, Chomel on, 109; lumbar, Dr. Perkins' case of, 146; of the lung, 312
Address, Professor Bird's valedictory, 54
Alumina, sulphate of, 63, 112
Abnormal sounds in the ear, 81
Ammonia, muriate of, in hemicrania, 12; in scirrhus of the stomach, 94
Amputation, mortality after, 249
Aneurism of the aorta, Dr. Pepper's case of, 4; opening into lungs, 118; dissecting of aorta, Michener's case of, 277
Anatomy, Wistar's Pancoast's notice of, 23; Horner's 232
Aneurism, false, Liston's case of, 42; statistics of Hunter's operation for, 84; popliteal, cured by compression of the femoral artery, 176; of the thigh, treated by pressure, Liston's case, 308
Ankylosis, false, at elbow-joint, Professor Mütter's case of, 51
Accidents in coal mines, 84
Air, introduction of, into veins, 95
Anemia of the brain induced by fatal hemorrhage, 129
Adulteration of common salt at Paris, 245
Apoplexy, meningeal, 132
Accoucheur's, French, 312
Acetic extracts, 146
Abortion, frequency of criminal, 247
Ash, Dr. J. W., his case of fatal perforation of the duodenum, 169
Anatomical atlas, Smith's, 269
Advantage of medicine in a liquid form, 312
Austria, Wilde on the medical institutions of, 170
Arteries, structure of, 94
Anal fistula, statistics of, 177
Antagonism of phthisis and intermittent fever, 227
Anatomy of the ganglionic nerves, 180
Arsenic, new test for, 212
Ascites, Dr. Denig's case of, 231
Aran, manual of diseases of the heart, by 232

B.

Barry, Dr. Martin, his observations on the development of blood corpuscles, 156
Belladonna as a prophylaxis in scarlet fever, 307
Bibliographical notices, 11, 23, 41, 42, 43, 54, 66, 68, 77, 78, 79, 87, 88, 104, 125, 126, 139, 140, 149, 162, 170, 174, 175, 198, 199, 209, 210
Blindness caused by vitriol, 46
Bigelow, Dr. J. B., his case of strangulation of the sigmoid flexure, 164
Board of naval surgeons, 50
Black cataract, 177
Bailey, Dr., case of hepatic colic, 49
Bruit de soufflet in anemia, cause of, 216
Bird, professor's, valedictory address, 54
Bridges, Dr. Robert, notice of his edition of Graham's chemistry, 224
Blood, physiology and pathology of, 129
Brain, anemia of, induced by fatal hemorrhage, 129
Burt, Dr. J. L., case of poisoning by laudanum, 146
Bichat, statue of, at Bourg, 244
Bronchitis, sputa in, 272
Bronchial blenorrhœa, 271
Burwell, clinical reports, by Dr. 294

C.

Carbonic acid, exhaled during respiration, 46; formed in typhus fever, 95; with quinine in intermittent fever, 120; exhalation of, from the body, 228
Cervical vertebra, Prof. Horner's case of luxation of, 3
Cataract, green, 55; peculiar appearances in, 72; black, 177
Caries of the teeth, cause of, 60
Caldwell, his physiology vindicated, 210
Chapped nipples, 75
Cancer, statistics of, 179; frequency of, 200; Walshe on, 261
Clinical lectures, 1, 5, 8, 13, 17, 19, 22, 25, 27, 30, 37, 39, 51, 61, 64, 73, 76, 85, 97, 103, 104, 109, 121, 135, 147, 157, 161, 193, 217, 229, 241, 253, 265, 281, 282, 289, 290, 292, 301
Cephalalgia, treatment of, 275
Condie, treatise on the diseases of children, by Dr. F. 297
Correspondence, foreign, 24, 50, 89, 225, 298
Chomel, clinical lectures, by 13, 25, 37, 61, 73, 85, 97, 109, 121, 157, 193, 205, 241, 265
Churchill, Dr. Fleetwood, his diseases of females noticed, 34; his midwifery noticed, 198
Columba root, 165
Cells, theory of opposed, 45
Climate in pulmonary consumption, 127
Colic, hepatic, Dr. Chomel's lecture on, 24; Dr. Bailey's case of, 49; lead, prevention of, 60
Cyanosis, Dr. Craigie on the pathological causes of, 300
Calculi in St. George's Hospital, 119
Clymer, Dr. Meredith, case of soft cancer of the ovary, 148; remarks on tubercular peritonitis by, 258; clinical lectures by, 282, 301

D.

Difference in the pus-like globules of the blood in health and disease, 48
Degeneration, fatty, of the arteries, 95
Dispensary, quarterly report of the obstetric department of, 99
Dispensatory, United States, notice of, 199
Diagnosis, physical, of diseases of the chest, 162
Delirium tremens, H. Johnson on, 117, 248

Derangement, mental, Dr. Willis on, 176
Diabetes Mellitus, Dr. Percy on, 215; importance of abstinence from bread in, 236
Deformities from burns, relief of cicatrices in, 236
Death of Dr. Jacobson, 244; of Dr. Harlan, 245
Druggist, narrow escape of, 245
Dissector, Goddard's, Wilson's notice of, 270
Diathesis, Hysteric, 273
Dunglison, contributions from, 63, 86, 208, 268

E.

Epilepsy, Dr. I. Parrish on, 14; statistics of, 211
Epulis, Dr. Mütter on, 27
English physicians, 58
Effects of climate on phthisis, 163
Ear, Pilcher on diseases of, 68
Elements of chemistry, Graham's, 224
Editorial remarks, 12, 69, 79, 91, 104, 115, 126, 140, 200
Ethics, Medical, 79, 115
Eruptions, syphilitic, 55
Extirpation of the parotid gland, 80; of a uterine polypus, 181
Experiments on the torpedo, 118
Electro-puncture, 96; in the treatment of deafness, 144; in hydrocele, 90
Effects of imprisonment on blacks, 259
External agencies on disease, influence of, 191
Expedition to the Niger, medical history of, 222
Exhalation of carbonic acid from the body, 228

F.

Fatty degeneration of the arteries, 95
Fever with pericarditis, 142; African remittent, 222
Foreign correspondence, 24, 89
Fracture of the clavicle without displacement, 35; of the lower end of radius, 59; of the femur, 82; of the skull, 133; compound, of leg, 155; statistics of, 192
False Aneurism, Liston's case of, 42
Fibrous polypus of the uterus, 144
False jalap, 56
Freezing, revival after, 60
Fistula lachrymalis, 145; anal, 177
Fissure of the soft and hard palate, 88
Function of the skin, 128

G.

Gallic acid in menorrhagia, 226
Gangrene of the lung, 262
Glanders, case of, in man, 84, 186
Galt's practical medicine, 269

H.

Harris, Dr. Chapin, dissertation on maxillary sinus by, 125; his address, 209
Health of the insane, influence of diet on, 95
Hæmospasy, 240
Hemp, Indian, 108, 271
Hernia, strangulated crural, 9; operation for, in an infant seven weeks old, 35; unique species of, 167
Hemicrania, muriate of ammonia in, 12; treatment of, 276
Harlan, death of Dr. 245
Horner, Dr., clinic of, 5
Hepatic colic, Chomel on, 25; Dr. Bailey on, 49
Hayward, Dr., on the statistics of pulmonary consumption, 34
Herpetic pruritus, 70
Hemiplegia from tying the carotid, 188, 205
Hemorrhages, uterine, 83; prognosis of, 216
Homeopathic literature, 126; results of, 155
Hooping cough, tannin in, 107
Huguier, lectures on diseases of females, 253
Hydriodate of mercury and arsenic, 80
Hysteria, Chomel on, 97; anomalous, 256
Hysteric diathesis, 273
Hydrocele, spermatozoa in the fluid of, 168; encysted, 185; Dr. Randolph on, 281

I.

Indian hemp, 108, 271
Iliac abscess, 109
Illegal practice of medicine at Paris, 132
Influenza, Dr. Tucker on, 159
Infusion, cold, of cinchona, 151
Indigestion, Dr. W. Philip on, 175
Iodide of potassium in atonic ulcers, 46
Iodurated syrup of sarsaparilla, 177
Iridoplasma, 36
Insanity, restraint in treatment of, 184; plea of, 204
Influence of season on pneumonia in children, 180; of asphyxia on secretion of bile, 156; of the seasons, 263
Iris, state of, in cerebral disease, 154
Intestines, case of strangulated, 164
Iodine, test for, in mineral water, 189; in un-united fracture, 275
Inflammation of the nervous centres, 288

J.

Jalap, false, 56
Johnston, Dr. W. Poyntell, clinical lectures by, 5, 17, 30
Johnston, Professor W. P., clinical lectures by, 292, 293

K.

Kidneys, Berzelius on, 140, 149
Kennedy on scarlet fever, 276

L.

Lectures by
Chomel, 13, 25, 37, 61, 73, 85, 97, 109, 121, 157, 193, 205, 241, 265
Clymer, 282, 301
Dr. W. Poyntell Johnston, 5, 17, 30, 99
Prof. W. P. Johnston, 292, 293
Prof. Mütter, 8, 19, 27, 51, 64, 76, 104
Prof. Mitchell, 161, 147
Prof. Pancoast, 9, 21, 291
Roux, 1, 217
Huguier, 253
Rostan, 229
Randolph, 281
Leaming, Dr. F., case of needle lodging in heart by, 112
Liquor taraxaci, 12
Lactucarium, 45
Lactate of quinine, 47
Lead colic, prevention of, 60
Luxations of cervical vertebra, 5, 17
Liquor of hydriodate of mercury and arsenic, 80
Lithotrity and lithotomy in children, 276

M.

Materia medica and therapeutics, Pereira's elements of, 87
Magnetisers, 7; hint to, 152
Maxillary sinus, Harris on diseases of, 125
Marsh, Sir Henry, on regurgitation, 186
Menstruation, physiology of, 44; effects of on secretion of milk, 185
Meningeal apoplexy, 132
Meigs', letter on puerperal fever, from Dr. 3
Malaria, contributions to history of, by Prof. Dunglison, 268
Medical clinic, Andral's, 162
profession, 69
ethics, 79, 115
officers of the navy, 91, 104, 140
McCormac on prolapsus ani, 184
McDowell on curability of phthisis, 139
Mollities ossium, 178
Mitchell, clinical lectures by, 147, 161

Mental derangement, 176
Medicine, Williams' principles of, 286
Mortality of the plague, 69; influence of seasons on, 179; after amputation, 249, 263
Minor surgery, Dr. H. H. Smith on, 198
Menorrhagia, gallic acid in, 226
Malarious fevers, cause of, 208
Mitral valves, disease of, 107
Muriate of ammonia in hemicrania, 12; in scirrhus of the stomach, 94
Mütter, clinical lectures by, 8, 19, 27, 51, 64, 76, 104, 135, 289
Mineral acids in dropsy, 70
Midwifery, Churchill's theory and practice of, 198
Mercury, spurious preparations of, 298

N.

Naptha, 246, 255, 262
Naval surgeons, 50, 91, 104, 140
Nervous opthalmia, 58
Nipples, chapped, 75
Nitric acid in hemorrhoids, 66
Naphthaline, 108
Nutgalls, extract of, 108

O.

Officers, medical, of the navy, 91, 104
Ointment of nutgalls, 108
Ophthalmia, nervous, 58; gonorrhœal, 239
Opodeldoc, formula for, 273
Ovarian dropsy, cases of, 148, 192
Oxalic acid, 216

P.

Pancoast, clinical lectures by, 9, 21, 291
Parrish, Dr. Isaac, case of epilepsy by, 14; clinics of, 39
Pepper, case of aneurism of the aorta, by Dr., 4
Pereira, elements of materia medica by, 87
Parotid gland, extirpation of, 80
Paraplegia, 215
Perforation of the duodenum, 169
Paralysis of the bladder, 180
Paracentesis cerebri, Dr. Pancoast's case of, 291
Polypus of the nose, 103, 104, 135; of the uterus, 141, 181
Physiology of menstruation, 44; of the blood, 129; vindicated, 210; Carpenter's human, 221
Pennock, case of typhoid fever, by Dr, 123
Pneumonia, 73; in children, 150; treatment of, 151; prognosis in, 152
Plague, mortality of, 69
Phthisis pulmonalis, Dr. Wilson's case of, 86; influence of climate on, 127
Principles and practice of medicine, Elliotson's, 260; Williams', 286
Pruritus, herpetic, 70
Peritonitis, strumous, 131; tubercular, cases of, 257; Clymer on, 258
Prout on renal diseases, 206
Pyelitis, 166
Puerperal fever, Meigs and Rutter on, 3; Chomel on, 61
Pus-like globules of the blood, 48
Physicians, English, 58
Pulse, comparative frequency of, morning and evening, 154

Q.

Quarterly report of the obstetric department of the Philadelphia dispensary, 97
Quinine in large doses in typhus fever, 72; in rheumatism, 156; in traumatic fever, 178
Quackery, suppression of, 113
Quarterly summary of the Philadelphia college of physicians, 126

R.

Ranula, chemical composition of the fluid of, 59; operation for, 76
Radius, fractures of lower end of, 59
Renal disease simulating calculus in the bladder, 96
Revival after freezing, 60; of the arts amongst the Greeks, 120
Ricord, lecture on syphilis by, 152; on the treatment of gonorrhœa, 202
Roux, clinical lectures by, 1
Restraint in the treatment of insanity, 184
Remittent fever, African, 222
Regurgitation, Sir H. Marsh on, 186
Rickets, influence of, on the growth of the skull, 214
Reptiles, circulation of the blood in, 215
Rostan, clinical lecture by, 229
Ruptures, Lawrence's treatise on, 78
Rupture of the uterus, recovery from, 239

S.

Saffron, medical use of, 214
Scrophula, 39
Sulphuretted hydrogen gas as a cause of fever, 208
Stewart, hospitals of Paris, by Dr. F. Campbell, 149
Small-pox, Dr. Gregory on, 311
Spermatozoa in the fluid of hydrocele, 168
Salivation, treatment of, 108
Strumous peritonitis, 131
Scarlatina, Dr. Kennedy on, 276
Scirrhus of the stomach, 94
Stammering, 118
Spinal irritation, case of, 243
Structure of the arteries, 94
Sputa in bronchitis, 272
Suette miliare, 44
Sudden deaths at Strasbourg, 46
Surgery, Lisfranc's clinical, 11; Fergusson's practical, 79; minor, by Dr. H. H. Smith, 198
Strangulated crural hernia, 10; hernia, successful operations for, 35, 44; intestines, 164, 280
Sulphate of alumina, 63, 112
Syphilis, Ricord on, 152; Clymer on, 282
Syphilitic eruptions, 55
Somnambulic dogs, 151
Stomatitis ulcerosa, 216

T.

Taraxaci, liquor, 12
Taraxacum, cream of, 276
Tannin in hooping cough, 107
Taliacotian operation, 56
Tænia, statistics, 163
Tavernier, lever-belt and busk for spinal deformities, by Dr., 288
Tinea favosa, treatment of, 155
Toynbee on the local causes of deafness, 203
Treatment of pneumonia, 151
Test for arsenic, 212
Traumatic fever, quinine in, 178
Tubercular peritonitis, Dr. Clymer on, 158; cases of, 157
Tucker, case of abscess, by Dr. D. H., 74
Tubercles in serous membranes, 117; seat of, 312
Turning of plants towards the light, 156
Typhus fever, quinine in, 72; carbonic acid formed in, 95
Typhoid fever, case of, by Dr. Pennock, 123; amongst animals, 156

U.

University of Pennsylvania, 38; clinic of, 17, 30
Unhealthiness of Liverpool, and its causes, 227
Urethroplasty, 120
Upshur, case of catalepsy, by Dr. G. L., 243
Urea in the blood in pneumonia, 179

Ununited fracture, treated by iodine, successfully, 275
Uterus, fibrous polypus, 144, 181; venous inflammation, 238; irritable, 270
Uterine diseases, diagnosis of, 211; hemorrhages, 83
Urine, sugar in gouty, 240; in morbus Brightii, 263; in diseases of spinal cord, 264; in pneumonia, 273

V.

Varicocele, 47
Varicose veins, 99
Varus mentagra, 247
Venereal diseases, Ricord on, 34; Clymer on, 282, 301
Veins, introduction of air into, 95
Vital statistics of Sheffield, 272
Vesico-vaginal fistula, 71, 142

W.

Wallace, clinical reports, by Dr. Ellerslie, 113, 138, 304
Will's hospital, clinic at, 39
Wilson, case of phthisis, by Dr., 86; on diseases of the skin, 104; on nervous disorders, 189
Wistar, system of anatomy, by Dr. Caspar, 23
Willis, mental derangement, 170
Warren, Dr. J. Mason, taliacotian operation by, 56; on fissure of the palate, 88; on cancer, 261
Wenzel the oculist, 175

### THE MEDICAL EXAMINER,

### And Retrospect of the Medical Sciences.

EDITED BY

MEREDITH CLYMER, M.D.

*Editorial Remarks*, . . . . . . . . . 12

Muriate of Ammonia in Hemicrania, . . 12

Liquor Taraxaci, . . . . . . . . . 12

Published every alternate Saturday, and subscriptions received, by J. C. TURNPENNY, N. E. corner of Spruce and Tenth streets. Price, Three Dollars a year, invariably in advance. Two copies sent to the same address, Five Dollars.

Communications and Letters to be addiessed (always pre-paid) to the Editor of the Medical Examiner, Philadelphia.

Postmaster's franks can always be obtained for remittances.

This journal is subject to newspaper postage only.

### PROSPECTUS.

### THE MEDICAL EXAMINER.

EDITED BY

MEREDITH CLYMER, M. D.

The sixth volume of this Journal is commenced on this day, (January 21, 1843,) and will be issued punctually every *alternate* Saturday. It is devoted to the subjects usually embraced in a medical journal, viz., Original Communications, Clinical and other Lectures, Hospital Reports, Reviews and Notices of all New Publications, Editorial discussion of topics involving Medical Politics and Ethics, and a Summary of American and European Improvements in Medicine, condensed from the various Periodicals. During the *five* years which have elapsed since the commencement of the Examiner, Lectures and Papers have appeared from the most eminent Professors and Practitioners in this City, and in different sections of the Union.

A series of letters from correspondents in Paris has been given, and arrangements have been made for their continuation. It will be the aim of the Editor to make the Examiner a practical record of the progress of the Medical Sciences, securing, by its short intervals of publication, the rapid dissemination of novelties, presented at frequently recurring intervals, and in a readable amount of matter.

The Medical Examiner is published punctually every *alternate* Saturday, at *Three Dollars* per annum, *invariably* in advance. Address, *post-paid*, (Post Masters' franks can always be had for remittances,) the Editor of the Medical Examiner, Philadelphia.

Subscriptions received at the Office, N. E. corner of Spruce and Tenth streets.

☞ This No. is sent as a specimen.

